# Effect of dexmedetomidine priming on convulsion reaction induced by lidocaine

**DOI:** 10.1097/MD.0000000000004781

**Published:** 2016-10-28

**Authors:** Xi-Feng Wang, Xiao-Ling Luo, Wei-Cheng Liu, Ben-Chao Hou, Jian Huang, Yan-Ping Zhan, Shi-Biao Chen

**Affiliations:** a Department of Anesthesia, the First Affiliated Hospital of Nanchang University, Nanchang; b Department of Anesthesia, the Seventh People’ s Hospital of Shenzhen, China.

**Keywords:** convulsion, dexmedetomidine, excitatory amino acids, inhibitory amino acids, lidocaine

## Abstract

To study the effect of dexmedetomidine priming on convulsion reaction induced by lidocaine.

The New Zealand white rabbits were applied for the mechanism study of dexmedetomidine priming for preventing convulsion reaction induced by lidocaine. The influence of dexmedetomidine priming with different doses on the time for convulsion occurrence and the duration time of convulsion induced by lidocaine, as well as contents of excitatory amino acids (aspartate [Asp], glutamate [Glu]) and inhibitory amino acids (glycine [Gly], γ-aminobutyric acid [GABA]) in the brain tissue were investigated.

With 3 and 5 μg/kg dexmedetomidine priming, the occurrence times of convulsion were prolonged from 196 seconds to 349 and 414 seconds, respectively. With dexmedetomidine priming, the contents of excitatory amino acids (Asp, Glu) were much reduced at occurrence time of convulsion comparing with that without dexmedetomidine priming, while content of inhibitory amino acids Gly was much enhanced.

The application of dexmedetomidine before local anesthetics can improve intoxication dose threshold of the lidocaine, delay occurrence of the convulsion, and helped for the recovery of convulsion induced by lidocaine. The positive effect of dexmedetomidine on preventing convulsion would owe to not only the inhibition of excitatory amino acids (Asp, Glu), but also the promotion of inhibitory amino acids Gly secretion.

## 1 Introduction

Lidocaine, since Swedish chemist Lofgren firstly synthesized it in 1943, has been widely applied in the fields of clinical anesthesia, pain therapy (e.g., the trigeminal neuralgia), as well as treatment of respiratory diseases (e.g., asthma), nervous system diseases (e.g., infantile convulsion, epilepsy, and vertigo), digestive system diseases (e.g., hiccough), and arrhythmia, because of its good pharmacological action. However, excessive application of lidocaine or infiltration into blood vessels can result in local anesthetics poisoning. The central nervous system toxicity convulsion induced by local anesthetics is a common clinical complication. Early symptom usually exhibited excitation, such as lip tongue numbness, headache, blurred vision, tinnitus, aphasia, etc., and without treatment would developed into central nervous system inhibition, circulatory collapse, and even respiratory stop, coma, seriously threating to life safety.^[1]^

Previous studies have demonstrated that benzodiazepines or barbiturates as preanesthetic medication were benefit for the prevention and reduction of local anesthetics toxicity. Improving the safety threshold value of lidocaine and prevention the convulsion reaction induced by lidocaine are important clinical problems urgently to be resolved. The pentothal sodium and midazolam are conventional anticonvulsants. The pentothal sodium has strong vascular stimulation and significantly inhibition effect on respiration and circulation, while midazolam would result in respiratory depression and aggravating hypoxia when quickly injected. Moreover, high dose using midazolam could cause ataxia. Hence, new anticonvulsant with less side-effect should be developed.

As a highly selective central agonist of α_2_-adrenergic receptors, dexmedetomidine is an anxiolytic, sedative, and analgesic medication, with advantages of mild influence on respiration, stress response inhibition, nonirritating, and nerve protective effect.^[2]^ Dexmedetomidine is notable for its ability to provide sedation without risk of respiratory depression (unlike other commonly used sedatives such as propofol, fentanyl, and midazolam) and can provide cooperative or semiarousable sedation.^[^3–8^]^

The current study suggests that application of dexmedetomidine before local anesthetics can reduce the occurrence of local anesthetics poisoning.^[9]^ Animal experiment results show that application of dexmedetomidine can improve intoxication dose threshold of the bupivacaine.^[10]^ In 2005, Tanaka et al^[11]^ firstly reported that dexmedetomidine can reduce the incidence of bupivacaine and levobupivacaine induced convulsion, as well as the toxicity of local anesthetics. Du et al^[12]^ reported that intravenous pregiven dexmedetomidine can significantly improve the threshold dose of bupivacaine intoxication, delay occurrence of cardiac toxicity, and alleviate the rats with bupivacaine induced cardiac toxicity with dose-dependent manner in a certain range. However, up to now, there is rare contribution devoted to the effect of dexmedetomidine priming on convulsion reaction induced by lidocaine.

In this work, the influence of dexmedetomidine priming with different doses on the convulsion time of the New Zealand white rabbits induced by lidocaine, as well as contents of excitatory amino acids (aspartate [Asp], glutamate [Glu]) and inhibitory amino acids (glycine [Gly], γ-aminobutyric acid [GABA]) in the brain tissue was investigated. And the preliminary action mechanisms were discussed.

## 2 Experimental

### 2.1 Animals

A total of 40 male New Zealand white rabbits (Wanqianjiaxing Co. Whhan) with weight of 2 to 2.5 kg were housed as standard protocols and feeding in clean environment, free drinking, and eating. The temperature maintained in 22 to 24 °C, and the relative humidity 50% to 60%.

The rabbits were randomly divided into 4 groups with 10 pregroup.

1. Group D1: normal saline priming and lidocaine injection for convulsion.
2. Group D2: 3 μg/kg dexmedetomidine priming and lidocaine injection for convulsion.
3. Group D3: 5 μg/kg dexmedetomidine priming and lidocaine injection for convulsion.
4. Group C: normal saline.

### 2.2 Protocols

The ethics committee of the First Affiliated Hospital of Nanchang University approved this study. The white rabbits were anesthetized by intraperitoneal injection of 40 mg/kg 1% sodium pentobarbital. Auricular vein catheters were set and fixed. Then the white rabbits were fixed in the prone position in the operating table, shearing the head villus and sterilization operation area. After longitudinal incision of the skin and subcutaneous tissue (about 1.5 cm) and exposed skull, the operating point selection sited at the middle line about 0.3 cm distance near the occipital protuberance. Drill hole in the direction of the 45° and epidural catheter to the direction of the foramen magnum. Clear cerebrospinal fluid flows along the tube. Cut-off at 5 cm from the skull, and connected to the epidural catheter injection head. Suture the skin and subcutaneous tissue, and fixed the catheter and the rabbit hair. Right thigh shearing, femoral artery catheter placed with heparinized saline heparin (20 mg/L). The depth was about 2 to 3 cm. The catheter was fixed to the surrounding tissue, and then the distal end of the catheter sealed with heparin cap.

After 24 hours, the white rabbit was fully conscious. Then the microsyringe pumps were connected onto auricular vein for continuous infusion of drugs. For group D1, 8 mL 0.9% normal saline were pumped in 10 minutes, then 2% lidocaine was pumped with the speed of 4 mg/kg/minute, and stopped immediately when convulsion. For Group D2, 8 ml 0.9% normal saline containing 3 μg/ kg dexmedetomidine was pumped and then pumped lidocaine in similar manner. Group D3: 8 ml 0.9% normal saline containing 5 μg/ kg dexmedetomidine was pumped and then pumped lidocaine in similar manner. For group C, 0.9% normal saline was pumped with average liquid dosages of group D1, D2, and D3. The white rabbits were all mask for oxygen during the drug infusion, and the dead ones were eliminated from the experiment.

The times for convulsion occurrence (t) and the duration times of convulsion (tt) of the rabbits in groups D1, D2, and D3 were recorded. The recovery of convulsion was determined by the time that the white rabbits could self-stand up.

For white rabbits in groups D1, D2, and D3, when they were recovery from convulsion, 2 mL arterial blood was pumped out from the femoral artery, stationary on the ice for 1 hour and then centrifugation for 10 minutes. The plasma was stored in −20 °C refrigerator. The lidocaine content in plasma of each group was measured by high efficiency liquid chromatography.

The 0.5 mL cerebrospinal fluid was collected at the times of drill catheter (T0), convulsion occurrence (T1), and 30 minutes after convulsion (T2). The cerebrospinal fluid put on the dry ice for freezing and then centrifuged for 20 minutes (4 °C, 13,000 r/minute). The supernatant was stored in −20 °C refrigerator. The contents of excitatory amino acids (Asp, Glu) and inhibitory amino acids (Gly, GABA) in the cerebrospinal fluid were tested by reversed phase high performance liquid chromatography fluorescence (RP-HPLC) method.

### 2.3 Statistical analysis

All statistical analyses were performed using SPSS version 13.0 (SPSS Inc., Chicago, IL). Measurement data were expressed as the mean ± SD and compared with single factor analysis of variance among groups. A *P* value of less than 0.05 was considered statistically significant.

## 3 Results and discussions

**Figure 1:**
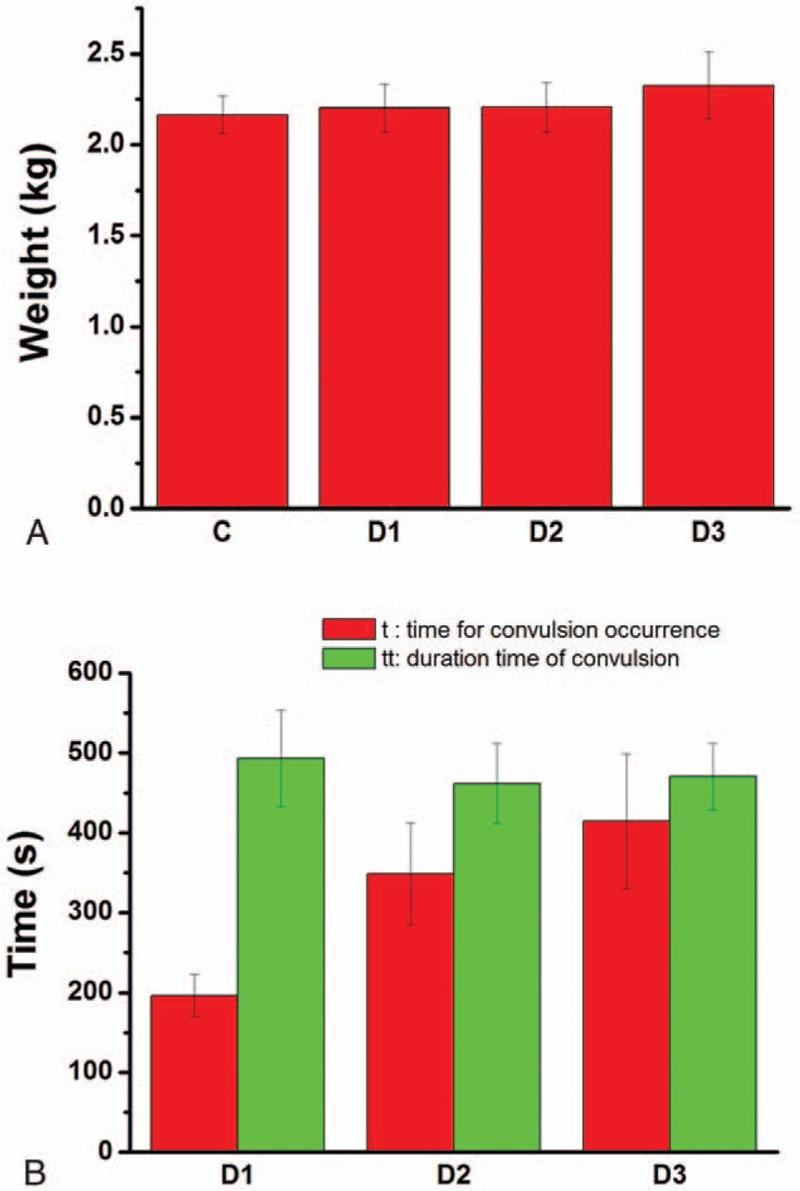
(A) The weights of the white rabbits in each group; (B) the times for convulsion occurrence (t) and the duration times of convulsion (tt) of the rabbits in groups D1, D2, and D3.

Figure 1A shows the weights of the white rabbits ranged in 2.0 to 2.5 kg. The weight showed no statistically significant difference among the groups. The times for convulsion occurrence (t) as well as the duration times of convulsion (tt) of the rabbits in groups D1, D2, and D3 were shown in Fig. 1B. For group D1, the lidocaine injection without dexmedetomidine priming, the average occurrence time of convulsion was about 196 seconds after beginning of lidocaine injection. With 3 μg/kg dexmedetomidine priming in group D2, the occurrence time of convulsion was prolonged to 349 seconds. When the primed dexmedetomidine increased to 5 μg/kg, the occurrence time was ever prolonged to 414 seconds, indicating a dexmedetomidine depended of occurrence time of convulsion. The results were statistically significant. Hence, it can be concluded that the dexmedetomidine priming can improve intoxication dose threshold of the lidocaine and delay occurrence of the convulsion induced by lidocaine. The duration time of convulsion was defined from the occurrence time of convulsion to the time that the white rabbits could self-stand up. The duration times of convulsion (tt) in group D1, D2, and D3 were 493, 462, and 471 seconds, respectively. The duration time was slightly reduced by dexmedetomidine priming. Considering that the total amount of lidocaine in the white rabbits with dexmedetomidine priming (group D2, D3) was much larger than that in group D1 because of the postponing of convulsion, the dexmedetomidine could help the rabbits get recovery from deeper lidocaine poisoning in fewer time. The results suggest that application of dexmedetomidine before local anesthetics had obvious positive effect for preventing the lidocaine induced convulsion.

**Figure 2:**
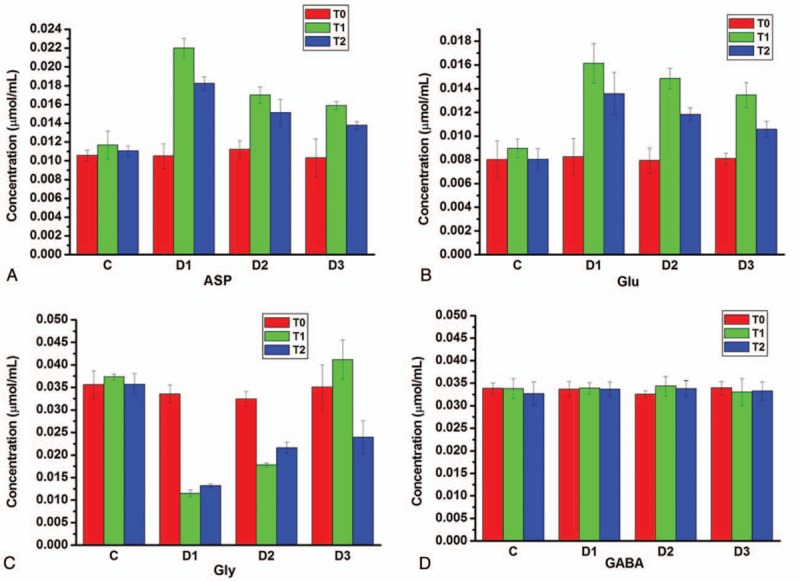
(A) The contents of Asp in the cerebrospinal fluid at the times of drill catheter (T0), convulsion occurrence (T1), and 30 minutes after convulsion (T2); (B) The contents of Glu in the cerebrospinal fluid at T0, T1, and T2; (c) The contents of Gly in the cerebrospinal fluid at T0, T1, and T2; and (d) the contents of GABA in the cerebrospinal fluid at T0, T1, and T2. Asp = aspartate, GABA = γ-aminobutyric acid, Glu = glutamate, Gly = glycine.

To explore the mechanism of dexmedetomidine priming for the prevention of convulsion reaction induced by lidocaine, the contents variation of excitatory amino acids (Asp, Glu) and inhibitory amino acids (Gly, GABA) in the cerebrospinal fluid at the times of drill catheter (T0), convulsion occurrence (T1), and 30 minutes after convulsion (T2) were tested. It can be seen in Fig. 2A that the asparagic acid (ASP) in the cerebrospinal fluid of each group at the times of drill catheter (T0) had no significant difference. At the time of convulsion occurrence, the Asp content of cerebrospinal fluid in Groups D1 without dexmedetomidine priming was much enhanced more than 1 times from 0.0105 to 0.022 μmol/mL. At 30 minutes after convulsion, the content of Asp was reduced to 0.018 μmol/mL. As an excitatory amino acid, the content of Asp in cerebrospinal fluid was directly related to the procedure of convulsion, and the occurrence of convulsion would attribute to the over secretion of the Asp induced by lidocaine injection. With dexmedetomidine priming, the Asp over secretion was obviously inhibited after lidocaine injection. Moreover, the larger amount of dexmedetomidine priming could result in stronger inhibition effect. Hence, dexmedetomidine would prevent the convulsion by controlling the Asp level in cerebrospinal fluid. The variation of Glu, the other excitatory amino acids, had the nearly similar regular pattern to that of Asp. The secretion of Glu could be much enhanced by lidocaine injection but depressed within dexmedetomidine priming, as shown in Fig. 2B. It is consistent with the previous report that the release of Glu could be inhibited by Dexmedetomidin via the evocation of K^+^ channel blocker 4-aminopyridine.^[13]^ One of the main mechanisms of convulsion induced by lidocaine is the NMDA-Ca^2+^-NO signaling pathway. The central inhibitory neurons would be inhibited by local anesthetics lidocaine, resulting in the dominant of excitatory neuron. The excitatory amino acid Glu released by neuronal was increased and the receptor channel was opened. In condition of the dexmedetomidine, presynaptic α_2A_ adrenoceptors are participated inhibition of release. With chelating of extracellular Ca^2+^ ions and dexmedetomidine associating with the vesicular transporter inhibitor bafilomycin A1, the Glu release was blocked. Via Ca_v_2.2 (N-type) and Ca_v_2.1 (P/Q-type) channels,^[14]^ the Ca^2+^ entered into the cell along the concentration gradient and activated calmodulin, which had effect on nitric oxide synthase (NOS). Excitatory amino acid, NMDA, Ca^2+^, and NO had interaction and reciprocal causation mediated convulsion. As the increasing ratio depolarization-induced intrasynaptosomal Ca^2+^ levels would be prevented, inhibitory effect dexmedetomidine would be abolished by blocking the Ca_v_2.2 and Ca_v_2.1 channels. Moreover, Glu release was also promoted by mitogen-activated/extracellular signal-regulated kinase (MEK) inhibitors via effect of dexmedetomidine.^[15]^ Hence, as α_2A_ adrenoceptors, the inhibitory effect of dexmedetomidine on the Glu release was directly associated with the blocking of voltage-dependent Ca^2+^ channels, kinase activity of mitogen-activated protein, as well as NMDA-Ca^2+^-NO signaling pathway.^[13]^ Our data were consistent with these previous studies.

Figure 2C showed the contents of inhibitory amino acid Gly in the cerebrospinal fluid at T0, T1, and T2. In group D1, the content of Gly was greatly reduced from 0.034 to 0.011 μmol/mL when convulsion occurrence. After stopping lidocaine injection, the Gly slightly recovered to 0.013 μmol/mL, indicating that lidocaine could block the secretion of the inhibitory amino acid Gly and thus induce the convulsion. With 3 μg/kg dexmedetomidine priming in group D2, the Gly content was increased at times T1 and T2 comparing with that in group D1. For group D2 with 5 μg/kg dexmedetomidine priming, the Gly content was much enhanced at the convulsion occurrence time from original 0.035 to 0.041 μmol/mL and 30 minutes later fallback to 0.024 μmol/mL with stopping lidocaine. These results suggested that the dexmedetomidine priming helped the secretion of Gly for preventing the convulsion. Larger amount dexmedetomidine would induce Gly over secretion, which could be re-regulated by the endocrine system of the body. For another inhibitory amino acid GABA, both lidocaine and dexmedetomidine seem to have no obvious influence on its secretion, as shown in Fig. 2D. The GABA content of cerebrospinal fluid in group D1 showed no significant variation at T0, T1, and T2. In group D1 and D2, the GABA content had also no statistically significant difference with dexmedetomidine priming, indicating GABA had not participated to the convulsion procedure.

In summary, application of dexmedetomidine before local anesthetics can improve intoxication dose threshold of the lidocaine, delay occurrence of the convulsion, and helped for the recovery of convulsion induced by lidocaine. The positive effect of dexmedetomidine on preventing convulsion would owe to not only the inhibition of excitatory amino acids (Asp, Glu), but also the promotion of inhibitory amino acids Gly secretion. This study provides more safe and effective therapy and theoretical basis for clinical prevention and treatment of anesthetics poisoning by lidocaine.
